# Designing and synthesizing perovskites with targeted bandgaps *via* tailored descriptors

**DOI:** 10.1039/d5sc04813c

**Published:** 2025-08-19

**Authors:** Kenshin Shibata, Fernando Garcia-Escobar, Tomoya Tashiro, Lauren Takahashi, Keisuke Takahashi

**Affiliations:** a Department of Chemistry, Hokkaido University North 10, West 8 Sapporo 060-0810 Japan fgares@sci.hokudai.ac.jp keisuke.takahashi@sci.hokudai.ac.jp; b List Sustainable Digital Transformation Catalyst Collaboration Research Platform, Institute for Chemical Reaction Design and Discovery, Hokkaido University Sapporo 001-0021 Japan

## Abstract

Descriptors that govern the bandgaps of perovskite-type oxides are identified by analyzing experimentally reported materials, focusing on compositional, structural, and electronic features relevant to solar energy conversion. These descriptors form the basis of a machine learning model that predicts bandgaps across a wide chemical space. Several compositions with targeted optical properties are predicted and subsequently synthesized. Structural and optical characterization studies confirm the formation of the predicted phases and the bandgap. Thus, this work demonstrates that the descriptor-driven, data-guided workflow accelerates the discovery of photoactive perovskites for solar energy conversion and visible-light-driven applications.

## Abstract 2

Descriptors for representing bandgaps of perovskite materials are identified using machine learning and literature data, and predicted perovskite materials for solar energy applications are synthesized experimentally.

## Graphical abstract

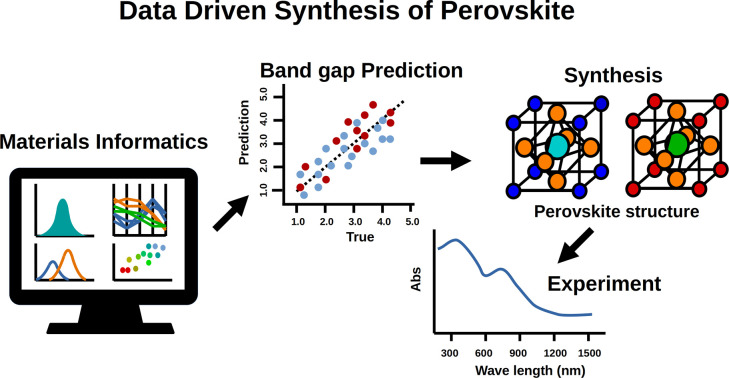

## Introduction

Designing materials with targeted properties presents a fundamental challenge due to the vast compositional space and the complex interplay between atomic structure, crystal symmetry, and synthesis conditions.^1–3^ Material properties such as bandgap are highly sensitive to subtle variations in atomic arrangement and processing methods, making rational design nontrivial. Trial-and-error searches of materials often struggle to efficiently navigate this multidimensional design space. The rise of materials informatics addresses this complexity, where machine learning and data mining are utilized to uncover the hidden patterns and trends for predicting materials properties.^4–7^ Such methodologies are particularly effective in perovskite materials, a class known for their tunable optoelectronic and catalytic properties.^8–12^ Here, machine learning is employed to predict the bandgaps based on a dataset compiled from experimental literature, where predicted perovskite materials are synthesized experimentally.

Perovskite materials are widely recognized for possessing near-ideal bandgaps for efficient solar light absorption, making them strong candidates for photovoltaic and photocatalytic applications.^13–16^ However, the bandgap in perovskites is highly sensitive to variations in atomic arrangement, including compositional tuning. If machine learning can unveil the hidden relationship between bandgap and atomic arrangement as well as descriptors for representing bandgap, one can consider that machine learning can be utilized to design the desired perovskite material.^9,17,18^ In this work, machine learning models are constructed using curated literature data to identify key material descriptors that govern bandgap behavior in perovskite materials. In addition, this work aims to link data, machine learning, experimental synthesis, and characterization in a unified discovery process. This approach provides insight into the underlying factors that control bandgap variation and offers a pathway for designing perovskites with tailored properties through informatics-guided screening.

## Methods

### Dataset collection and curation

The dataset used in this work is compiled from previous research on photocatalytic water splitting and contains multiple entries for the same material measured under different experimental conditions. It contains 540 data points from 151 papers.^19^ To focus on the relationship between the composition of materials and their bandgap, entries of perovskites with identical compositions but tested under different experimental conditions are removed to keep only one data point per unique perovskite composition. Therefore, 282 data entries are retained for further analysis.

This compiled dataset comprises the following variables: Ref, the reference number of the original publication; A, B, and X, the host elements in the perovskite lattice; A1, A2, B1, B2, and X1, the dopant elements at the corresponding sites; A mole through X1 mole, the weight fractions of these elements; Prep Meth, the synthesis method; CalcT, the calcination temperature (°C); Calc, the calcination time (hour); Prom Meth, the promoter loading method; Promoter, the elements used as the promoter; Prom, the weight percent of the promoter; Crystal, the crystal structure; BandGap, the optical bandgap energy (eV).

To uniquely identify each perovskite composition, a custom standardized label in the form of “ABX1.0_1.0_3.0” is used, where the numbers represent the normalized mole fractions of the A-site, B-site, and X-site elements, respectively. This label is not chemically meaningful but serves solely for data deduplication and analysis. To generate these labels, the molar amount (*n*_*i*_) is calculated as presented in eqn (1), where *w*_*i*_ is the weight fraction of element *i*, and *M*_*i*_ is the atomic mass of element *i*:1
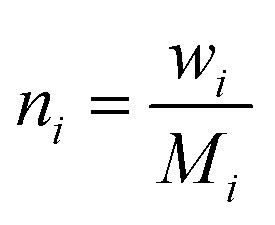


Next, the mole fraction for each site is calculated using eqn (2), where *n*_A_, *n*_B_, and *n*_X_ are the total molar amounts of elements at the A-, B-, and X-sites, respectively.2
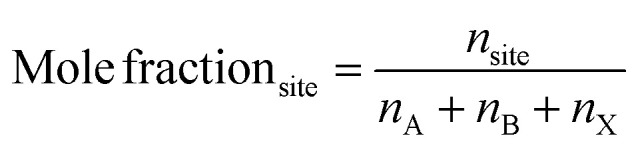


### Descriptor engineering

To construct machine learning descriptors from the perovskite composition data, a set of 754 base compositional descriptors is derived from elemental properties (*e.g.*, atomic volume) and the mole fractions of the constituent elements. These descriptors are generated by integrating site-specific compositions with 58 elemental property reference values obtained from the XenonPy library,^20^ and their types and calculation methods are summarized in Table 1. These descriptors aim to capture structure–property relationships in multi-site crystalline materials by incorporating both atomic-scale features and compositional heterogeneity.

**Table 1:** Proposed descriptor design for the bandgap prediction model for perovskite materials

Descriptor type	Captured information
*P*_*x*_	A property *P* value (*e.g.*, atomic volume) directly derived from the property *P* of the main element *x* occupying a specific crystallographic site *n* (A, B, or X) in the structure
*x* ∈ {Y, Cr, La…}	
*P*_*n*+*m*_ = *P*_*n*_ + *P*_*m*_	The sum of the property *P* values of the two main elements located at different crystallographic sites *n* and *m*. Used to capture synergistic effects between different sites
*n*, *m* ∈ {A, B, X}	
*P*_*n*−*m*_ = |*P*_*n*_ − *P*_*m*_|	The absolute difference of a property *P* between the main elements of two different sites *n* and *m*. Used to capture the contrast between elements at different sites
*n*, *m* ∈ {A, B, X}	
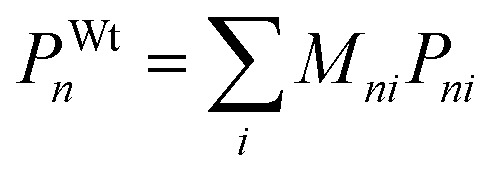	The weighted average of a given property *P* for all elements *i* at a single site *n*, accounting for partial occupancy or doping
*n* ∈ {A, B, X}, *i* ∈ {0, 1, 2}	
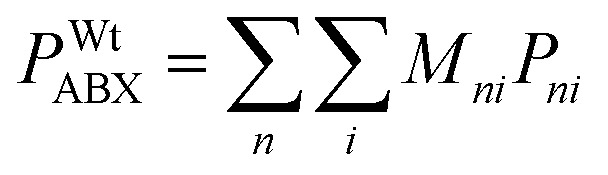	The weighted average of the properties *P* of all elements *i* across all sites *n* (A, B and X) representing an overall descriptor for the material
*n* ∈ {A, B, X}, *i* ∈ {0, 1, 2}	

To enhance the predictive performance of the machine learning model, additional descriptors are engineered by applying various mathematical transformations to the 754 base compositional descriptors. The applied transformations include power functions of degrees two to six, their corresponding roots, the natural logarithm, exponential functions, and reciprocals of all these transformations. As a result, a total of 9678 descriptors are generated.

### Descriptor selection and hyperparameter optimization

Support-Vector Regression (SVR) is implemented using the scikit-learn library^21^ to predict the bandgap of materials based on compositional descriptors, where SVR is chosen for its good performance on medium-sized datasets and its suitability for predicting continuous variables. Prior to descriptor selection, an initial tuning of the SVR hyperparameters is performed to determine appropriate values for the regularization parameter *C* and the kernel coefficient *γ*, with *ε* fixed at 0.1. For this purpose, 30 descriptors are randomly sampled from the set of engineered base compositional descriptors, and this process is repeated five times with different random seeds. For each trial, *γ* is first optimized by grid search over discrete candidates {2^−20^, 2^−19^, …, 2^10^}, selecting the value that maximizes the variance induced by a Gaussian kernel. Using the optimized *γ*, *C* is then selected from the same range by cross-validation as proposed by Kaneko *et al.*^22^ To further improve the model performance, the candidate ranges of *C* and *γ* are further refined through iterative grid searches around the initially selected values. Among the five trials, the best test *r*^2^ score of 0.61 is achieved with *C* = 3.86 and *γ* = 0.06, which are used in the subsequent descriptor selection step.

Descriptor selection is conducted using the MonteCat method proposed by Garcia-Escobar *et al.*,^23^ which is a Monte Carlo-based approach for identifying an optimal subset of descriptors that maximizes cross-validated prediction performance. The algorithm performs multiple optimization runs at different temperature settings, and the descriptor set yielding the highest cross-validation score across all runs is ultimately selected. In particular, the optimization process is repeated for 100 000 iterations in each run. A total of 35 independent runs are conducted, each using a different random seed and a distinct MonteCat temperature parameter value selected from the range 5, 10, 20, 50, 100, 200, and 500.

After identifying the optimal descriptor set with the Montecat algorithm, a final round of SVR hyperparameter tuning is performed using only the selected descriptors. This results in updated values of *C* = 4.25 and *γ* = 0.06, leading to an improved average test *r*^2^ score of 0.87. These values are employed in the final prediction model. All descriptor values are standardized using the StandardScaler module in scikit-learn, which centers each feature to zero mean and scales it to unit variance.

### Experimental synthesis

LaCrO_3_, LaFeO_3_, YFeO_3_, and YCrO_3_ are synthesized *via* a conventional solid-state reaction method using high-purity oxide precursors. For LaFeO_3_, 0.67 g of La_2_O_3_ (99.5% purity, Guaranteed Reagent, Junsei Chemical Co., Ltd.) and 0.33 g of Fe_2_O_3_ (Wako 1st Grade, FUJIFILM Wako Pure Chemical Corporation) are mixed and ball-milled at 400 rpm for 8 h using 20 g of zirconia balls in a planetary ball mill (ITO SEISAKUSHO, LP-M2). The milled powder is then transferred to an alumina crucible and calcined in air in a muffle furnace (KDF, 300 Plus). The calcination is performed by heating to 800 °C at a rate of 10 °C min^−1^, holding at 800 °C for 6 h, followed by cooling to room temperature over 4 h. YFeO_3_ is prepared under the same conditions as LaFeO_3_, using 0.59 g of Y_2_O_3_ (99.99% purity, FUJIFILM Wako Pure Chemical Corporation) and 0.33 g of Fe_2_O_3_ (Wako 1st Grade, FUJIFILM Wako Pure Chemical Corporation). For LaCrO_3_, 0.32 g of La_2_O_3_ (99.5% purity, Guaranteed Reagent, Junsei Chemical Co., Ltd.) and 0.15 g of Cr_2_O_3_ (Wako 1st Grade, FUJIFILM Wako Pure Chemical Corporation) are ball-milled for 16 h at 400 rpm using 20 g of zirconia balls. The milled powder is then calcined at 1100 °C at 10 °C min^−1^, held for 6 h, and cooled to room temperature over 4 h. YCrO_3_ is synthesized in the same manner as LaCrO_3_, using 0.22 g of Y_2_O_3_ (99.99% purity, FUJIFILM Wako Pure Chemical Corporation) and 0.15 g of Cr_2_O_3_ (Wako 1st Grade, FUJIFILM Wako Pure Chemical Corporation) as starting materials.

### Characterization methods

After synthesis, the phase composition, structural characteristics, and optical properties of the samples are analyzed using X-ray diffraction (XRD), scanning electron microscopy with energy-dispersive X-ray spectroscopy (SEM-EDS), and ultraviolet-visible-near-infrared (UV-vis-NIR) spectroscopy. X-ray diffraction (XRD) measurements are carried out using a diffractometer (MiniFlex600-C, Rigaku) equipped with Cu K*α* radiation (*λ* = 1.5406 Å), operated at 40 kV and 15 mA. The scan is performed in 1D mode over a 2*θ* range of 10–90° with a scan speed of 10.00° min^−1^ and a step width of 0.01°. These measurements are conducted to identify crystalline phases and to evaluate the phase purity and crystallinity of the synthesized materials. Elemental distributions are examined using scanning electron microscopy equipped with energy-dispersive X-ray spectroscopy (JCM-7000 NeoScope, JEOL). The powder samples are mounted on carbon tape without any conductive coating. Measurement conditions are as follows: accelerating voltage of 15.0 kV, working distance (WD) of 15.0 mm, magnification of ×1000, and high probe current (High-PC) mode under charge-reduction vacuum (CR Vac.) conditions. SEM-EDS elemental mapping is performed using a backscattered electron detector (BED-S) to assess the spatial distribution of constituent elements, focusing on the localization of A-site and B-site cations (*e.g.*, La/Fe, Y/Fe, La/Cr, and Y/Cr). Diffuse reflectance spectra in the UV-vis-NIR region are recorded using a spectrophotometer (V-770, JASCO). Measurements are performed over three wavelength ranges: 200–800 nm, 200–1000 nm, and 200–2700 nm. Optical bandgaps are estimated from Tauc plots derived from the reflectance data, assuming indirect allowed transitions.

### Materials prediction and candidate selection

Prediction of bandgap energies is conducted for 1852 perovskite-type compounds with the general formula ABO_3_, where the A- and B-site cations are selected from 60 candidate elements. The tolerance factor for each composition is calculated according to the method originally proposed by Goldschmidt, as described in ref. 24, using eqn (3), where *r*_A_, *r*_B_, and *r*_O_ denote the ionic radii of the A-site cation, B-site cation, and oxygen anion, respectively. Only compositions with tolerance factors between 0.7 and 1.0 are retained as geometrically feasible candidates.3
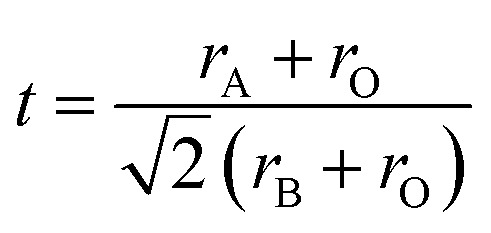


Using the optimized SVR model and selected descriptors, the bandgaps of these 1852 compounds are predicted. An inverse screening is conducted to identify candidates with chemically plausible structures and optical properties suitable for visible-light-driven solar energy applications, as detailed in the Results and discussion section. This screening yields 86 potential materials. Among these, four compounds LaCrO_3_, LaFeO_3_, YFeO_3_, and YCrO_3_ are selected for experimental validation.

## Result and discussion

### Data analysis and machine learning

**Fig. 1:**
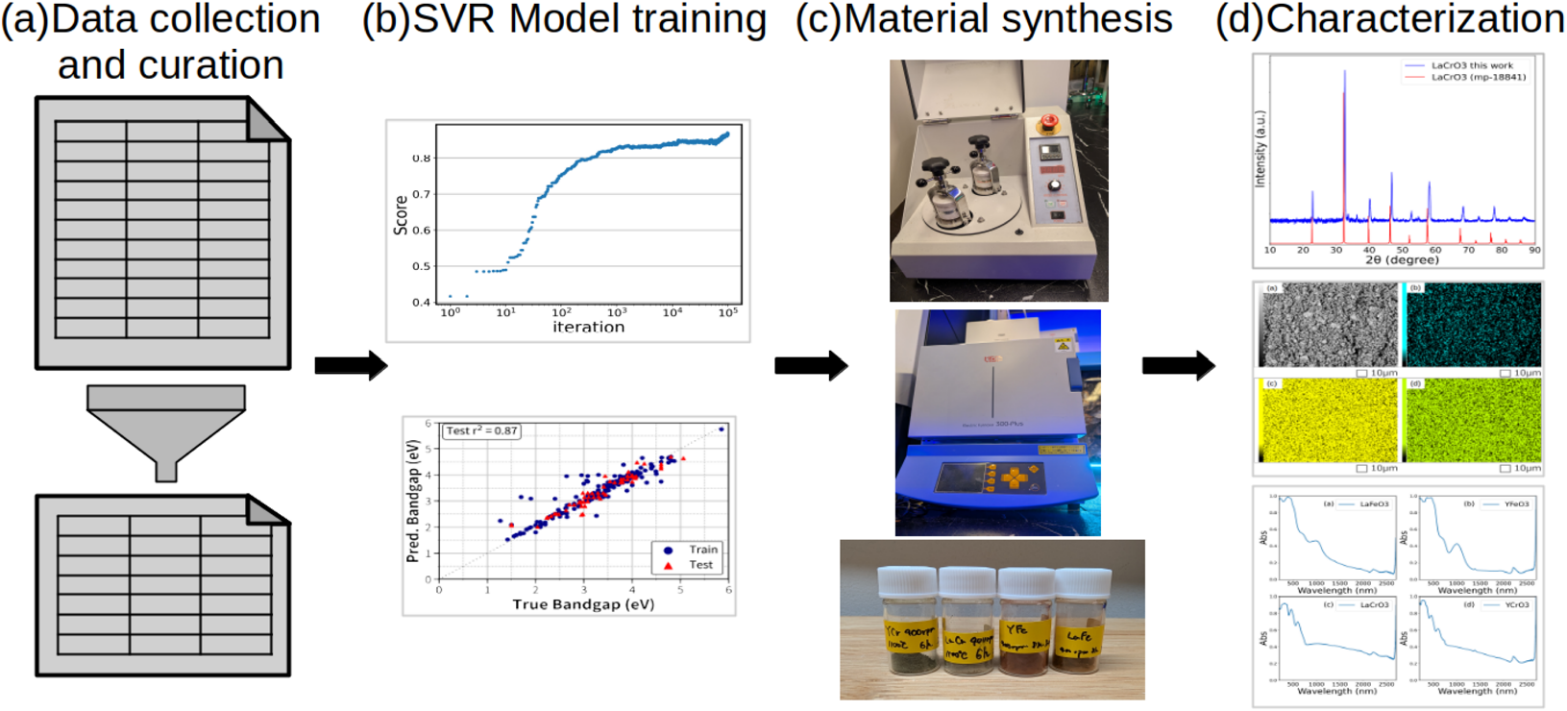
Proposed workflow for materials design. (a) Data collection and curation. (b) Machine learning and descriptor search. (c) Materials synthesis in the experiment. (d) Materials characterization.

The workflow for designing perovskite materials in this work is illustrated in Fig. 1. First, a literature dataset is collected, followed by data curation and preprocessing to generate a consistent and reliable dataset for further analysis. A machine learning model is then developed, incorporating feature engineering and descriptor selection using the MonteCat method to predict the bandgap in perovskite materials. Based on the predictions, perovskite materials with suitable bandgaps are identified. Among them, candidates are synthesized *via* solid-state reactions. The synthesized perovskite materials are characterized using XRD for structural analysis, SEM with EDS for morphology and elemental distribution, and UV-vis spectroscopy for bandgap estimation.

**Fig. 2:**
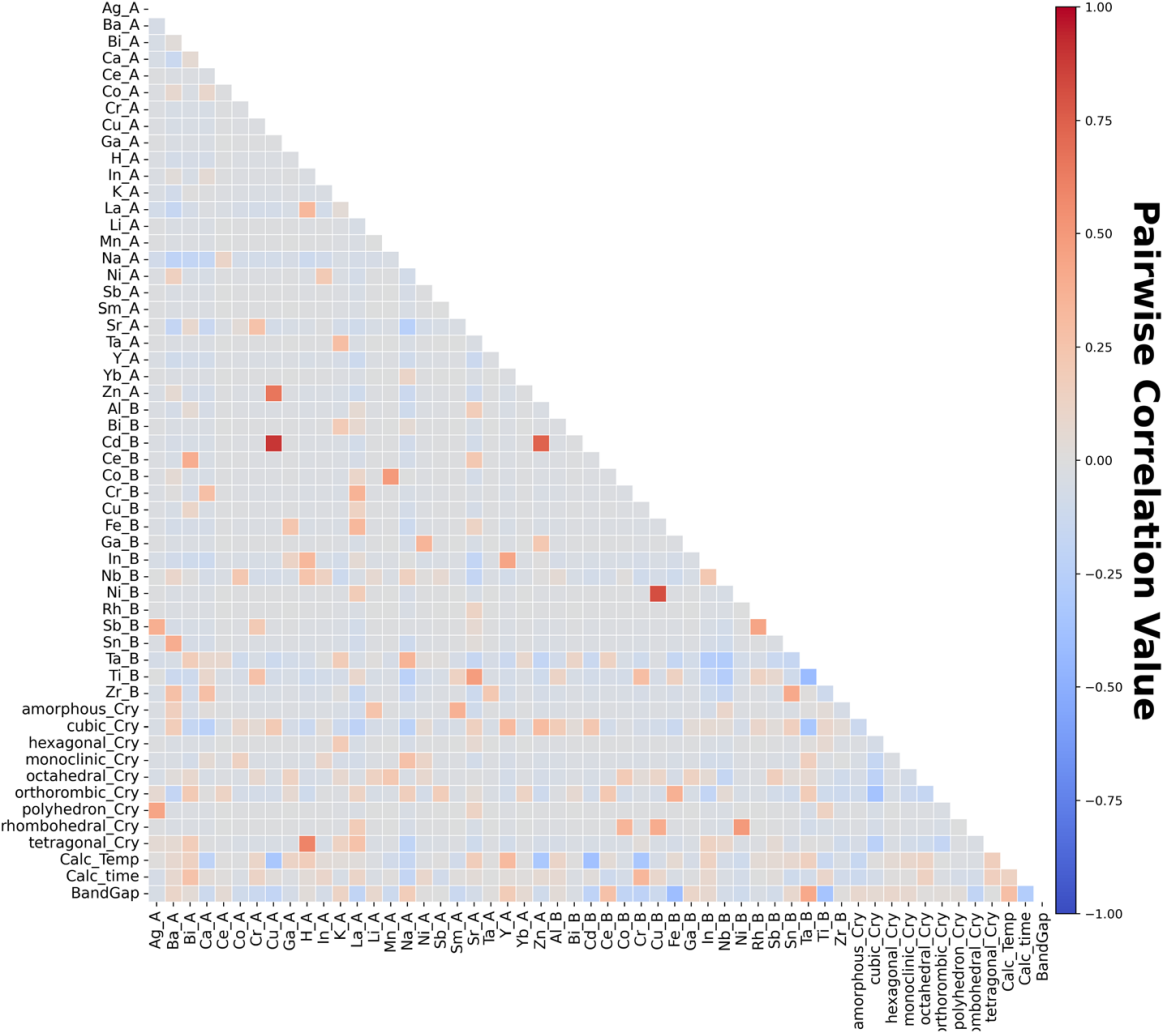
Pairwise correlation. A and B after the element indicate A-site and B-site, respectively.

Data analysis is performed in order to understand the statistical information. Pairwise correlation analysis is performed to examine the linear relationships among variables, as shown in Fig. 2. The chemical elements at the A and B sites are encoded using one-hot encoding. Categorical variables are thus transformed into binary features. The Calc prefix relates to variables in the calcination process: namely, calcination temperature (Calc_Temp in °C) and calcination time (Calc_time in h).

Fig. 2 indicates that the bandgap tends to decrease when the B-site element is one of the following: Cd, Co, Cr, Fe, or Ti. In contrast, it tends to increase when the B-site element is Ce or Ta. These tendencies suggest a notable influence of B-site elemental selection on the electronic structure of the materials. It is also noteworthy that bandgap tends to increase as the calcination temperature (CalcT) increases.

SVR with MonteCat is employed to identify the optimal set of descriptors for predicting the bandgap as a function of the elemental composition. As a result, 24 compositional descriptors are selected, as summarized in Table 2, along with their elemental property sources, descriptor construction types, and corresponding mathematical operations. Note that the descriptors identified in this work might, at least indirectly, reflect underlying physical properties such as the bandgap.

**Table 2:** Descriptors selected *via* MonteCat analysis

Property (*P*)	Descriptor (D)	Operation
num_p_unfilled	*P* ^Wt^_B_	e^−*D*^
covalent_radius_pyykko	*P*_A−B_	e^−*D*^
num_d_unfilled	*P*_A−B_	e^−*D*^
atomic_weight	*P* ^Wt^_A_	*D* ^6^
lattice_constant	*P*_B+X_	e^−*D*^
dipole_polarizability	*P* ^Wt^_B_	*D* ^2^
atomic_radius_rahm	*P*_B−X_	e^*D*^
atomic_radius	*P* ^Wt^_X_	*D* ^5^
atomic_volume	*P*_A+X_	*D* ^4^
electron_negativity	*P*_B−X_	*D* ^−3^
fusion_enthalpy	*P*_A−B_	e^*D*^
covalent_radius_cordero	*P*_A−X_	*D* ^3^
Density	*P* ^Wt^_ABX_	*D* ^−6^
hhi_r	*P*_A−B_	*D* ^5^
gs_bandgap	*P* ^Wt^_ABX_	*D* ^2^
num_d_valence	*P*_A−B_	e^*D*^
specific_heat	*P*_A_	*D* ^6^
num_p_valence	*P* ^Wt^_B_	*D* ^6^
melting_point	*P*_B_	*D*
covalent_radius_pyykko	*P*_A−B_	*D* ^3^
en_ghosh	*P* ^Wt^_A_	*D* ^4^
thermal_conductivity	*P*_A−X_	*D* ^6^
num_s_unfilled	*P* ^Wt^_B_	*D* ^5^
heat_of_formation	*P* ^Wt^_ABX_	*D* ^2^

**Fig. 3:**
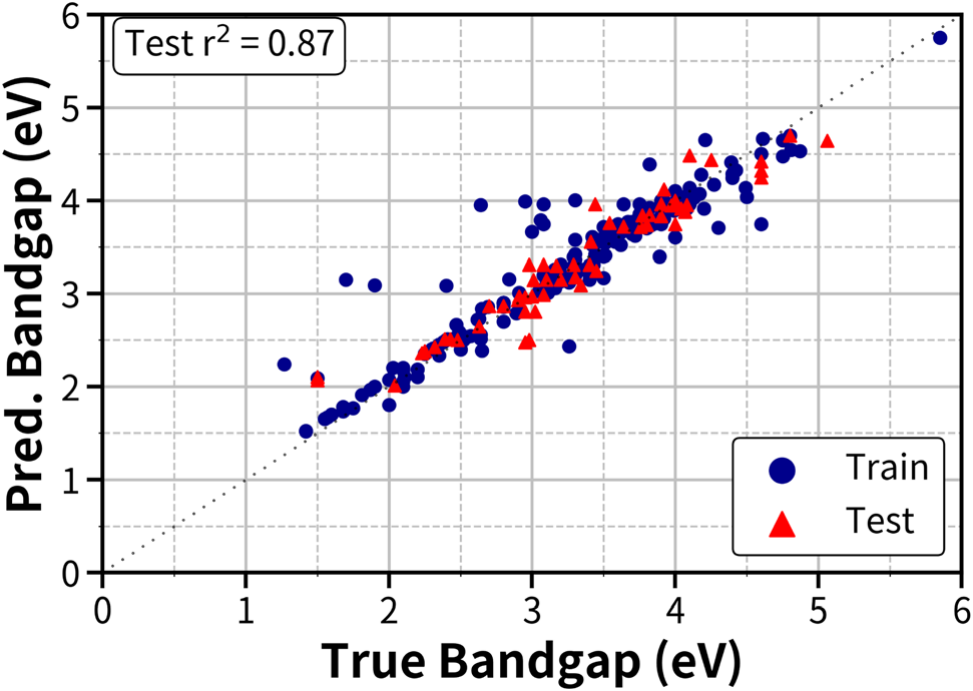
True *vs.* predicted bandgap using SVR.

With these descriptors, an SVR model resulted in an average test *r*^2^ score of 0.87, as shown in Fig. 3, where the *r*^2^ score is obtained by averaging the *r*^2^ scores over 10 random train-test splits (80% training and 20% testing).

Bandgap predictions are then conducted for 1852 hypothetical perovskite-type compounds with the general formula ABO_3_, where the A and B cations are selected from 60 elements including Li, Be, Na, Mg, Al, K, Ca, Sc, Ti, V, Cr, Mn, Fe, Co, Ni, Cu, Zn, Ga, Ge, Rb, Sr, Y, Zr, Nb, Mo, Tc, Ru, Rh, Pd, Ag, Cd, In, Sn, Sb, Cs, Ba, La, Ce, Pr, Nd, Pm, Sm, Eu, Gd, Tb, Dy, Ho, Er, Tm, Yb, Lu, Hf, Ta, W, Re, Os, Ir, Pt, Au, Hg, Tl, Pb, and Bi. The geometric feasibility of each composition is evaluated using the tolerance factor, calculated by eqn (3). Only combinations with tolerance factors in the range of 0.7 to 1.0 are retained for subsequent prediction analysis.

These 1852 compositions are then given to the trained SVR model to predict their corresponding bandgaps. Based on the predicted results, 86 candidates are selected according to the following two criteria: (i) the predicted bandgap lies between 0.45 eV and 2.2 eV, and (ii) the sum of the oxidation states of the A and B cations equals +6, assuming a perovskite-type ABO_3_ structure with charge neutrality. Among the 86 filtered candidates, four compounds LaCrO_3_, LaFeO_3_, YFeO_3_, and YCrO_3_ are selected for experimental synthesis based on their predicted bandgaps.

### Experimental results

#### X-ray diffraction (XRD) analysis

**Fig. 4:**
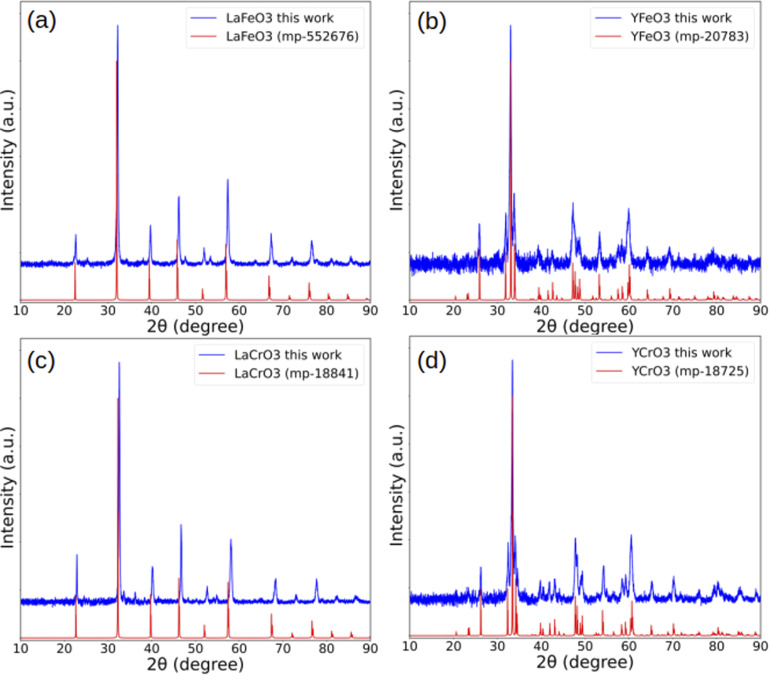
Comparison of experimental and simulated X-ray diffraction (XRD) patterns for the synthesized perovskite oxides: (a) LaFeO_3_, (b) YFeO_3_, (c) LaCrO_3_, and (d) YCrO_3_. Experimental patterns (blue) are shown along with simulated patterns (red) based on CIF files for the target compounds obtained from the Materials Project database.

Fig. 4(a)–(d) presents the X-ray diffraction (XRD) patterns of the synthesized LaFeO_3_, YFeO_3_, LaCrO_3_, and YCrO_3_ samples, along with the corresponding simulated patterns. The simulated patterns are generated using VESTA^25^ from CIF files obtained from the Materials Project^26^ database. The powder X-ray diffraction patterns are calculated by considering the Cu Kα doublet, comprising Kα_1_ (*λ* = 1.5405 Å) and Kα_2_ (*λ* = 1.5443 Å), with relative intensities of 1.0 and 0.5, respectively.

In all cases, the experimental XRD patterns closely match the simulated reference patterns of the target perovskite phases, with sharp and well-resolved peaks confirming the successful formation of the desired crystal structures. Notably, the peaks of LaCrO_3_ are slightly shifted to lower 2*θ* values compared to the simulated pattern. Note that the minor peaks observed in Fig. 4(c) and (d) are attributed to residual Cr_2_O_3_, indicating incomplete reaction during the formation of LaCrO_3_. These XRD results collectively confirm that the dominant crystalline phases in all samples are the intended perovskite structures, with no detectable secondary phases.

#### Electron microscopy (SEM) with elemental mapping (EDS)

**Fig. 5:**
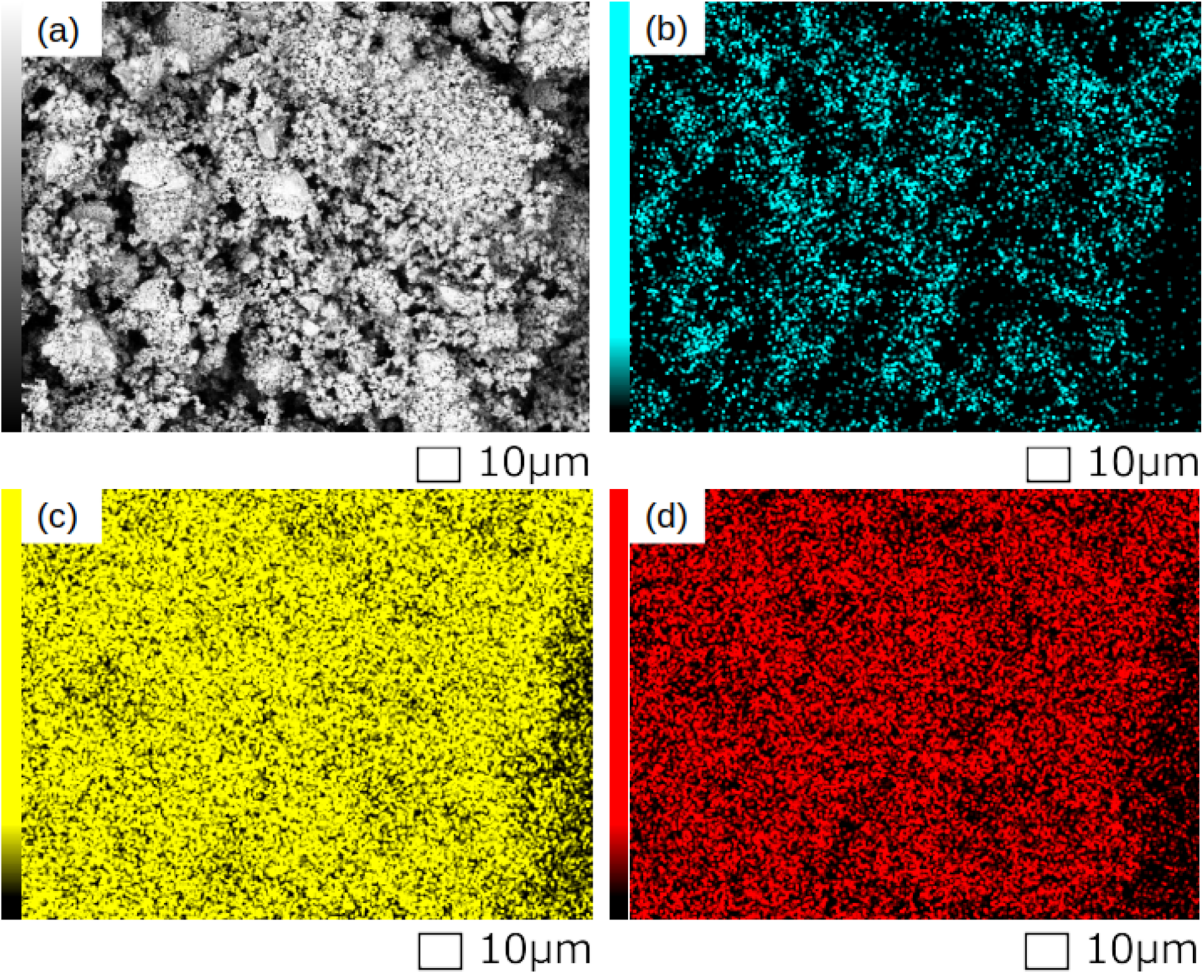
SEM image and elemental maps of LaFeO_3_ obtained by SEM-EDS. (a) SEM image, (b) O (K-line), (c) La (l-line), and (d) Fe (K-line).

To complement the structural information obtained from XRD, SEM-EDS elemental mapping is performed to evaluate the spatial distribution of constituent elements in the synthesized samples. Representative results for LaFeO_3_ are shown in Fig. 5, which includes the SEM image and corresponding elemental maps for O (K-line), La (L-line), and Fe (K-line). The elemental maps exhibit a high degree of spatial overlap among the constituent elements, indicating a homogeneous distribution of these elements throughout the sample. This homogeneous elemental distribution supports the formation of a single-phase perovskite structure, as identified by XRD analysis.

Similar elemental distribution patterns are observed for the other synthesized compositions (YFeO_3_, LaCrO_3_, and YCrO_3_), confirming that the A-site and B-site cations are homogeneously incorporated in each case without observable segregation. These results, in conjunction with the XRD analysis, provide strong evidence for the formation of single-phase perovskite structures with well-dispersed constituent elements. The full set of elemental maps for all samples is provided in the SI (Fig. S1–S3).

#### UV-vis spectroscopy and bandgap estimation

**Fig. 6:**
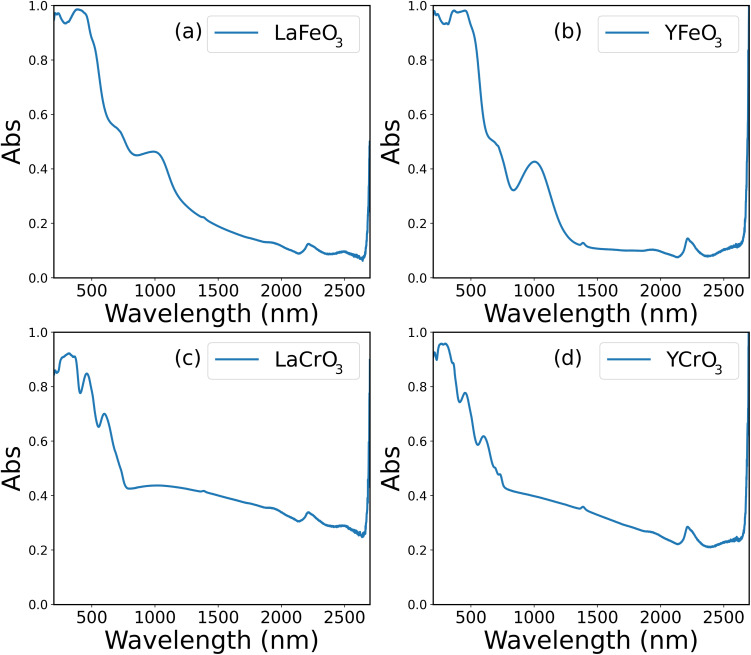
UV-vis-NIR absorption spectra of synthesized samples measured in the range of 200–2700 nm: (a) LaFeO_3_, (b) YFeO_3_, (c) LaCrO_3_, and (d) YCrO_3_.

Diffuse reflectance UV-vis spectroscopy is performed on LaFeO_3_, YFeO_3_, LaCrO_3_, and YCrO_3_ in the 200–2700 nm range, as shown in Fig. 6(a)–(d). The absorption features are primarily governed by the B-site cation: Fe-based samples exhibit broad absorption around 500 and 1000 nm, while Cr-based samples show sharper peaks near 330, 500, and 600 nm. These trends indicate that optical absorption properties are more strongly influenced by the B-site than the A-site element.

Optical bandgaps are estimated using Tauc plots, assuming indirect allowed transitions. Values obtained over the full spectral range (200–2700 nm) are 1.00 eV (LaFeO_3_), 1.04 eV (YFeO_3_), 1.71 eV (LaCrO_3_), and 1.64 eV (YCrO_3_). Reanalysis using narrower spectral windows (200–800 nm and 200–1000 nm) reveals notable shifts in estimated bandgaps (Table 3), particularly for samples exhibiting multiple overlapping absorption bands. The predicted bandgaps show good agreement with experimental values measured in the 200–800 nm range. Specifically, the synthesized LaFeO_3_, YFeO_3_, LaCrO_3_, and YCrO_3_ samples exhibited bandgaps of 2.16 eV, 2.13 eV, 1.90 eV, and 1.93 eV, respectively. These values closely match the model predictions of 2.09 eV, 1.95 eV, 2.15 eV, and 2.05 eV, with deviations within 0.2 eV. This agreement demonstrates that the model successfully captures patterns in the training data and is effective for guiding the synthesis of perovskite materials with target bandgaps. It must be noted that deviations appear when experimental data are extended beyond 1000 nm, where the model tends to overestimate the bandgaps. This discrepancy likely arises from the model's limited capacity to capture low-energy transitions, as such spectral regions are not well represented in the training data. These findings emphasize the importance of measurement range in evaluating optical properties and confirm the reliability of the model within the spectral window covered by the training data.

**Table 3:** Comparison of predicted and experimental bandgaps measured from UV-vis-NIR spectra over different wavelength ranges

Sample	Training data bandgap (eV)	Predicted bandgap (eV)	Experimental bandgap (eV)		
			200–800	200–1000	200–2700 nm
LaFeO_3_	2.10	2.09	2.16	1.79	1.00
YFeO_3_	N/A	1.95	2.13	1.73	1.04
LaCrO_3_	N/A	2.15	1.90	1.79	1.71
YCrO_3_	N/A	2.05	1.93	1.86	1.64

## Conclusion

A machine learning approach utilizing previously collected experimental data is employed to identify key descriptors governing the bandgaps of perovskite-type oxides and to predict their bandgaps. Based on the prediction results, several promising compositions are identified as potential photoactive materials for solar energy conversion and visible light driven applications. To validate the prediction, selected candidate materials are synthesized and subjected to structural and optical characterization. XRD analysis confirms the formation of the target perovskite phases, and UV-vis spectroscopy reveals that the experimentally measured bandgaps are in good agreement with the predicted values. These results highlight the effectiveness of combining descriptor-driven, data-guided prediction with experimental validation to accelerate the discovery of functional materials. The approach demonstrated here provides a practical framework for designing novel photoactive materials with targeted electronic properties, particularly in the context of solar energy conversion and visible-light-driven applications.

## Author contributions

K. T. conceived the central project idea. K. S., F. E., L. T., and K. T. performed data analysis and machine learning. K. S., T. T., and K. T. carried out experimental synthesis and characterization. K. T. and L. T. funded the project. All authors contributed to preparing the manuscript.

## Conflicts of interest

There are no conflicts to declare.

## Supplementary Material

SC-OLF-D5SC04813C-s001

## Data Availability

This study was carried out using the SI from E. Can and R. Yildirim, *Appl. Catal., B*, 2019, **242**, 267–283 at https://doi.org/10.1016/j.apcatb.2018.09.104. The supporting information provides a list of the descriptors used, the predicted materials, SEM images, and the data analysis and visualization. See DOI: https://doi.org/10.1039/d5sc04813c.
