# Supplementary material for: Designing and synthesizing perovskites with targeted bandgaps via tailored descriptors
Source: Chem Sci. 2025 Aug 19;16(36):16703–11. doi: 10.1039/d5sc04813c (PMC12362180; doi:10.1039/d5sc04813c)
Supplement: SC-016-D5SC04813C-s001 [file SC-016-D5SC04813C-s001.pdf]

Supplementary Information (SI) for Chemical Science.
This journal is © The Royal Society of Chemistry 2025

Supporting Information:

Designing and Synthesizing Perovskites with Targeted Bandgaps via Tailored Descriptors

Kenshin Shibata,† Fernando Garcia-Escobar*†, Tomoya Tashiro†,

Lauren Takahashi†, Keisuke Takahashi*†‡

Department of Chemistry, Hokkaido University, Sapporo, Japan

E-mail:fgares@sci.hokudai.ac.jp, keisuke.takahashi@sci.hokudai.ac.jp

Table S1: List of elemental descriptors used in this study

No.	Feature	Description
1	atomic_number	Number of protons in the nucleus of an atom
2	atomic_radius	Atomic radius
3	atomic_radius_rahm	Atomic radius reported by Rahm et al.
4	atomic_volume	Volume occupied by one mole of atoms
5	atomic_weight	Relative atomic mass
6	boiling_point	Boiling point at standard pressure
7	bulk_modulus	Measure of a substance's resistance to uniform compression
8	c6_gb	Dispersion coefficient (C6) from Grimme's method
9	covalent_radius_cordero	Covalent radius from Cordero et al.
10	covalent_radius_pyykko	Covalent radius from Pyykkö (single bond)
11	covalent_radius_pyykko_double	Covalent radius from Pyykkö (double bond)
12	covalent_radius_pyykko_triple	Covalent radius from Pyykkö (triple bond)
13	covalent_radius_slater	Covalent radius from Slater
14	density	Mass per unit volume
15	dipole_polarizability	Dipole polarizability
16	electron_negativity	General electronegativity
17	electron_affinity	Energy change when an electron is added
18	en_allen	Electronegativity based on Allen scale
19	en_ghosh	Electronegativity based on Ghosh scale
20	en_pauling	Electronegativity based on Pauling scale
21	first_ion_en	First ionization energy
22	fusion_enthalpy	Enthalpy change during melting

Continued on next page

No.	Feature	Description
23	gs_bandgap	Bandgap in the ground state
24	gs_energy	Ground-state energy
25	gs_est_bcc_latcnt	Estimated lattice constant for BCC structure
26	gs_est_fcc_latcnt	Estimated lattice constant for FCC structure
27	gs_mag_moment	Ground-state magnetic moment
28	gs_volume_per	Ground-state volume per atom
29	hhi_p	Herfindahl–Hirschman index based on production
30	hhi_r	Herfindahl–Hirschman index based on reserves
31	heat_capacity_mass	Specific heat capacity (mass basis)
32	heat_capacity_molar	Molar heat capacity
33	icsd_volume	Volume per atom from ICSD
34	evaporation_heat	Enthalpy of evaporation
35	heat_of_formation	Standard enthalpy of formation
36	lattice_constant	Lattice constant (typically cubic)
37	mendeleev_number	Mendeleev’s ordering number
38	melting_point	Melting point at standard pressure
39	molar_volume	Volume per mole of substance
40	num_unfilled	Number of unfilled orbitals
41	num_valance	Number of valence electrons
42	num_d_unfilled	Number of unfilled d orbitals
43	num_d_valence	Number of d valence electrons
44	num_f_unfilled	Number of unfilled f orbitals
45	num_f_valence	Number of f valence electrons
46	num_p_unfilled	Number of unfilled p orbitals
47	num_p_valence	Number of p valence electrons
48	num_s_unfilled	Number of unfilled s orbitals
49	num_s_valence	Number of s valence electrons
50	period	Period in the periodic table
51	specific_heat	Specific heat capacity
52	thermal_conductivity	Ability to conduct heat
53	vdw_radius	van der Waals radius
54	vdw_radius_alvarez	van der Waals radius from Alvarez et al.
55	vdw_radius_mm3	van der Waals radius (MM3 force field)
56	vdw_radius_uff	van der Waals radius from UFF
57	sound_velocity	Speed of sound in the element
58	Polarizability	Atomic polarizability

Table S2: Predicted Bandgaps of Perovskite-type Compounds

No.	Formula	A-site	B-site	X-site	Bandgap (eV)
1	PrCoO3	Pr	Co	O	1.489
2	DyCoO3	Dy	Co	O	1.492
3	ErCoO3	Er	Co	O	1.504
4	HoCoO3	Ho	Co	O	1.508
5	GdCoO3	Gd	Co	O	1.516
6	CeCoO3	Ce	Co	O	1.524
7	SmCoO3	Sm	Co	O	1.526
8	TbCoO3	Tb	Co	O	1.528
9	NdCoO3	Nd	Co	O	1.539
10	TmCoO3	Tm	Co	O	1.542
11	LuCoO3	Lu	Co	O	1.556
12	PmCoO3	Pm	Co	O	1.558
13	LaCoO3	La	Co	O	1.592
14	YCoO3	Y	Co	O	1.603
15	YbCoO3	Yb	Co	O	1.624
16	EuCoO3	Eu	Co	O	1.649
17	ScCoO3	Sc	Co	O	1.742
18	TmFeO3	Tm	Fe	O	1.769
19	BiCoO3	Bi	Co	O	1.804
20	TmCrO3	Tm	Cr	O	1.856
21	PrFeO3	Pr	Fe	O	1.868
22	ErFeO3	Er	Fe	O	1.882
23	DyFeO3	Dy	Fe	O	1.885
24	AuCoO3	Au	Co	O	1.890
25	GeCoO3	Ge	Co	O	1.917
26	SbCoO3	Sb	Co	O	1.919
27	TbFeO3	Tb	Fe	O	1.921
28	DyCrO3	Dy	Cr	O	1.927
29	SnCoO3	Sn	Co	O	1.934
30	ErCrO3	Er	Cr	O	1.934
31	CeFeO3	Ce	Fe	O	1.935
32	PrCrO3	Pr	Cr	O	1.940
33	YFeO3	Y	Fe	O	1.955
34	GaCoO3	Ga	Co	O	1.958
35	TiCoO3	Ti	Co	O	1.959
36	ScFeO3	Sc	Fe	O	1.964
37	HfCoO3	Hf	Co	O	1.968
38	PdCoO3	Pd	Co	O	1.969
39	GdFeO3	Gd	Fe	O	1.970
40	LuFeO3	Lu	Fe	O	1.973
41	HoFeO3	Ho	Fe	O	1.978
42	YbCrO3	Yb	Cr	O	1.986
43	SmCrO3	Sm	Cr	O	1.988
44	TbCrO3	Tb	Cr	O	1.988

No.	Formula	A-site	B-site	X-site	Bandgap (eV)
45	HoCrO3	Ho	Cr	O	1.991
46	YbFeO3	Yb	Fe	O	1.995
47	AlCoO3	Al	Co	O	1.999

Continued on next page

No.	Formula	A-site	B-site	X-site	Bandgap (eV)
48	SmFeO3	Sm	Fe	O	2.000
49	MnCoO3	Mn	Co	O	2.006
50	ZrCoO3	Zr	Co	O	2.009
51	PrTiO3	Pr	Ti	O	2.014
52	CeCrO3	Ce	Cr	O	2.033
53	GdCrO3	Gd	Cr	O	2.038
54	TbTiO3	Tb	Ti	O	2.039
55	TmTiO3	Tm	Ti	O	2.039
56	EuCrO3	Eu	Cr	O	2.048
57	LuCrO3	Lu	Cr	O	2.054
58	YCrO3	Y	Cr	O	2.054
59	PmCrO3	Pm	Cr	O	2.055
60	PmFeO3	Pm	Fe	O	2.062
61	NdCrO3	Nd	Cr	O	2.063
62	NdFeO3	Nd	Fe	O	2.064
63	TiFeO3	Ti	Fe	O	2.065
64	ScCrO3	Sc	Cr	O	2.066
65	VCoO3	V	Co	O	2.080
66	ErTiO3	Er	Ti	O	2.082
67	TlCoO3	Tl	Co	O	2.084
68	LaFeO3	La	Fe	O	2.087
69	GeFeO3	Ge	Fe	O	2.089
70	CrCoO3	Cr	Co	O	2.096
71	DyTiO3	Dy	Ti	O	2.100
72	AuFeO3	Au	Fe	O	2.102
73	HfZnO3	Hf	Zn	O	2.104
74	EuFeO3	Eu	Fe	O	2.107
75	TmVO3	Tm	V	O	2.133
76	HfFeO3	Hf	Fe	O	2.134
77	CeZnO3	Ce	Zn	O	2.139
78	InCoO3	In	Co	O	2.145
79	AlFeO3	Al	Fe	O	2.146
80	ZrZnO3	Zr	Zn	O	2.150
81	LaCrO3	La	Cr	O	2.154
82	HoTiO3	Ho	Ti	O	2.158
83	VFeO3	V	Fe	O	2.161
84	GeVO3	Ge	V	O	2.170
85	NdTiO3	Nd	Ti	O	2.180
86	ZrFeO3	Zr	Fe	O	2.196

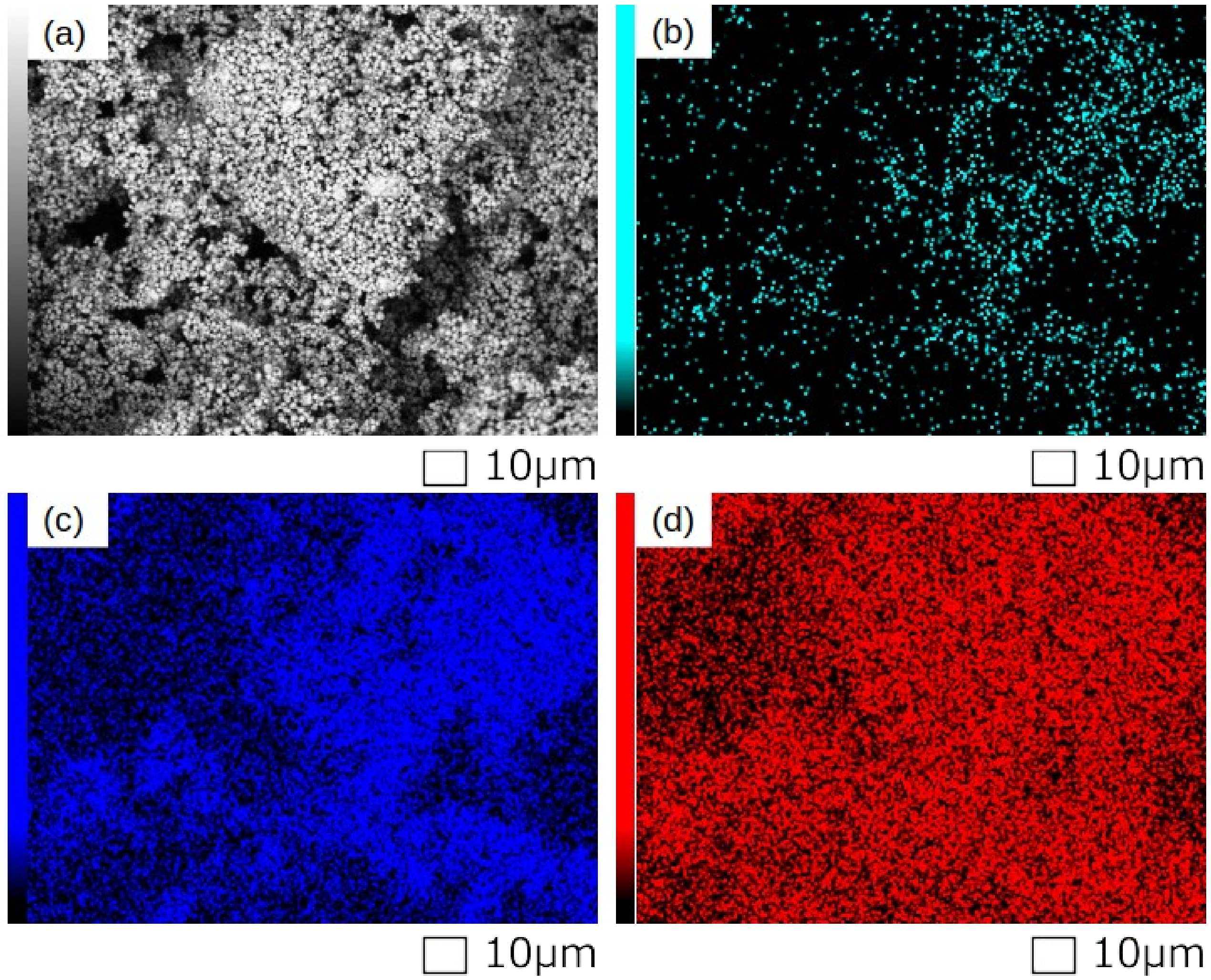

Figure S1: SEM image and elemental maps of $YFeO_3$ obtained by SEM-EDS. (a) SEM image, (b) O (K-line), (c) Y (L-line), and (d) Fe (K-line).

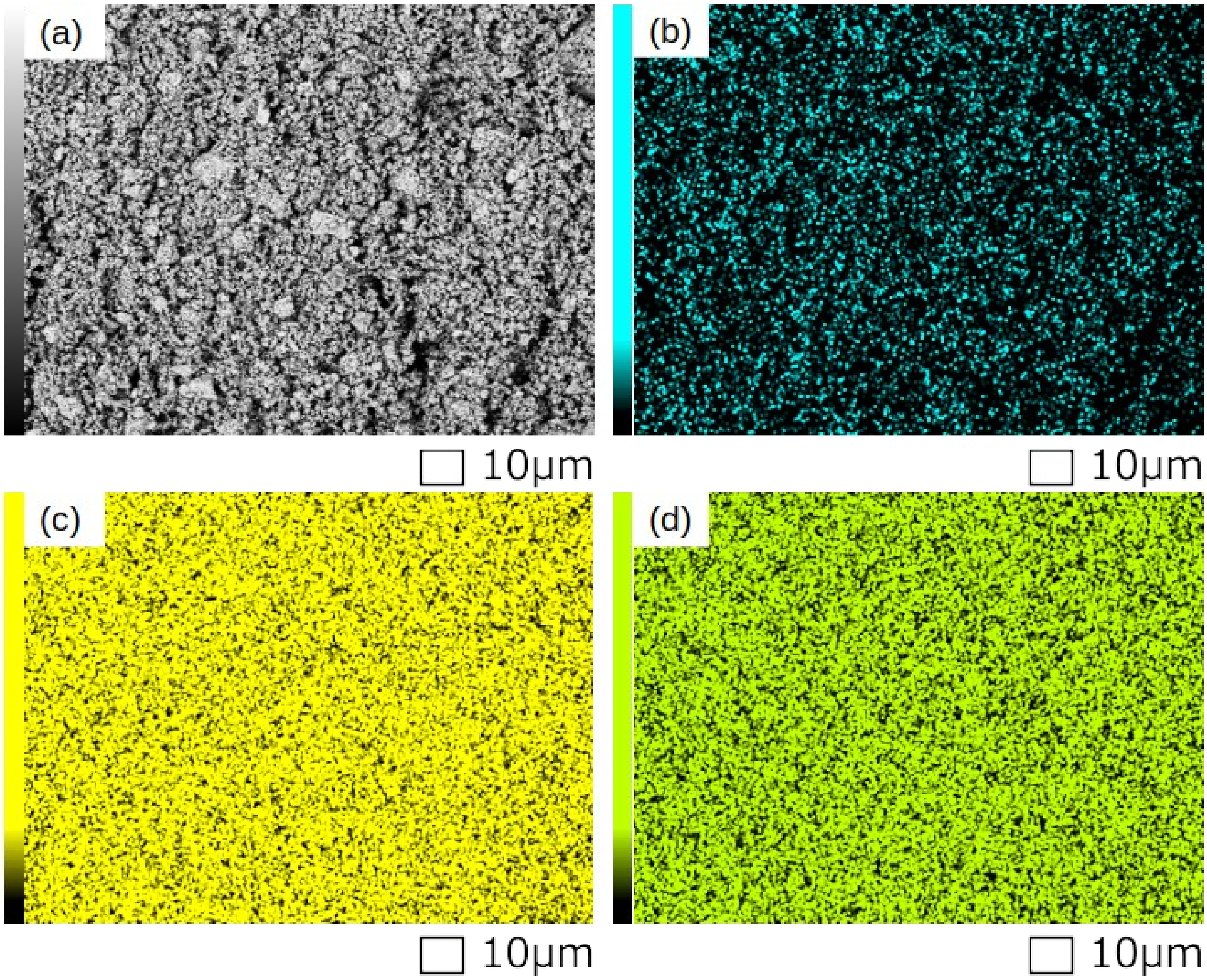

Figure S2: SEM image and elemental maps of $LaCrO_3$ obtained by SEM-EDS. (a) SEM image, (b) O (K-line), (c) La (L-line), and (d) Cr (K-line).

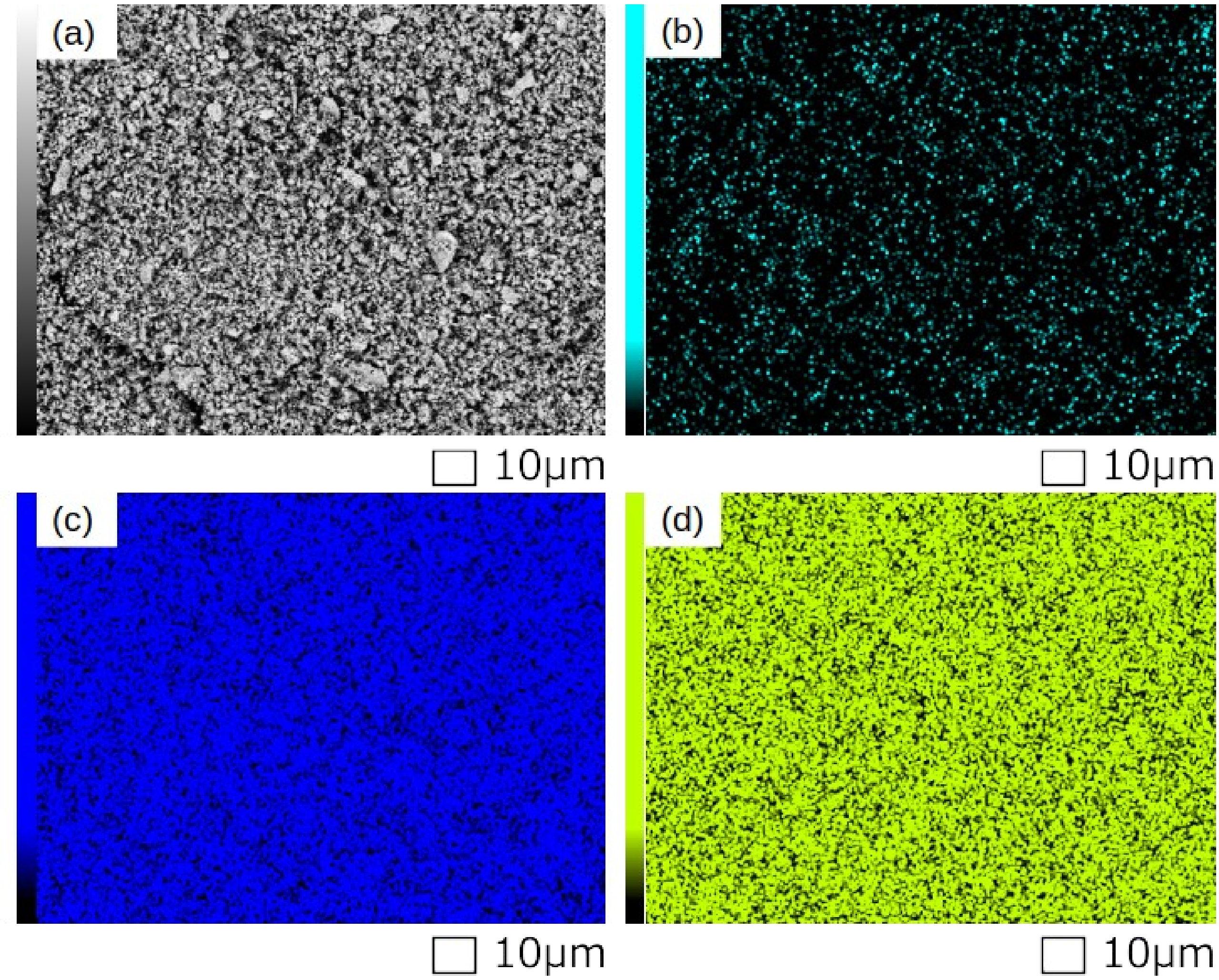

Figure S3: SEM image and elemental maps of $YCrO_3$ obtained by SEM-EDS. (a) SEM image, (b) O (K-line), (c) Y (L-line), and (d) Cr (K-line).

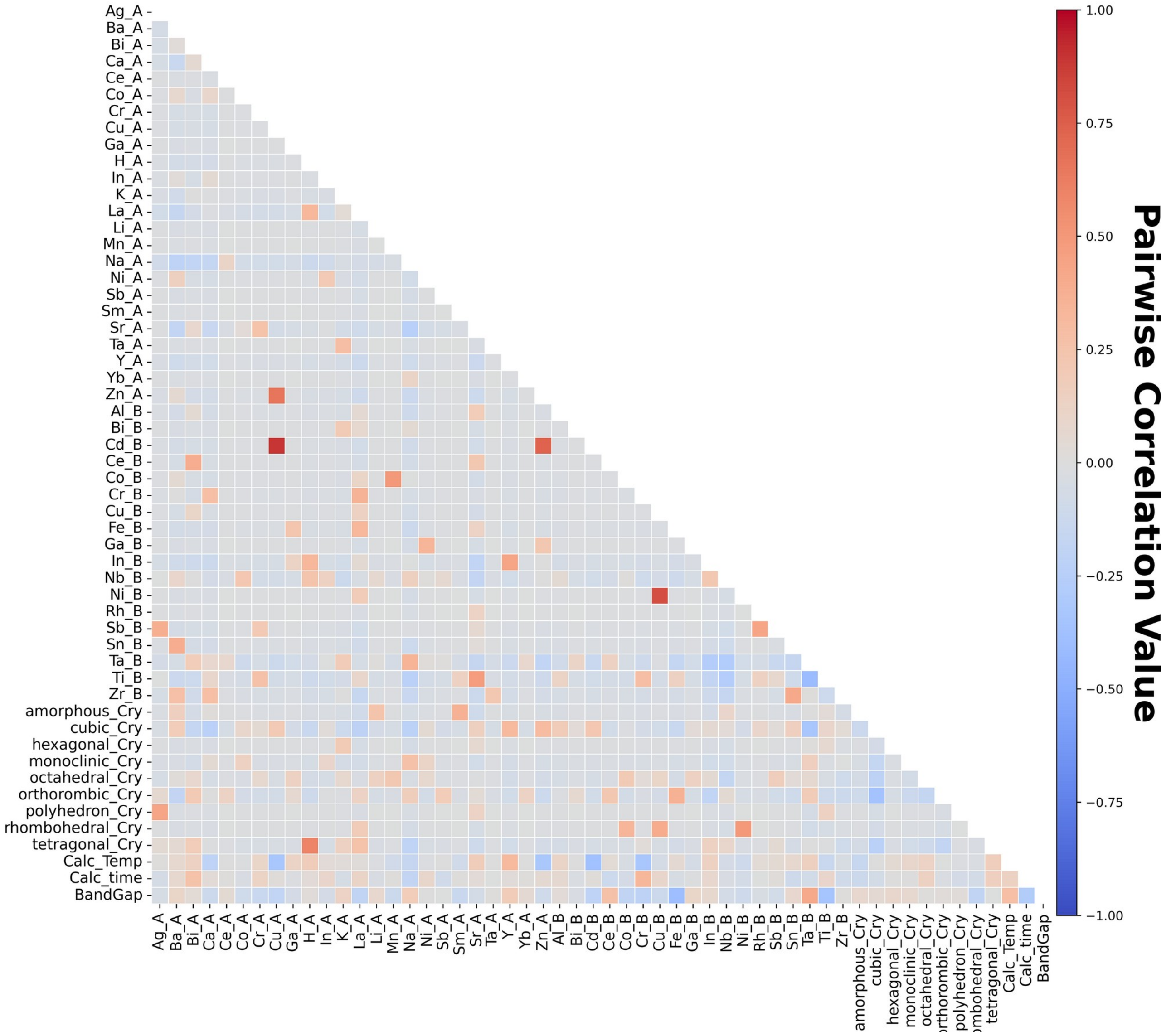

Figure 4S: Full scale version of Figure 1.

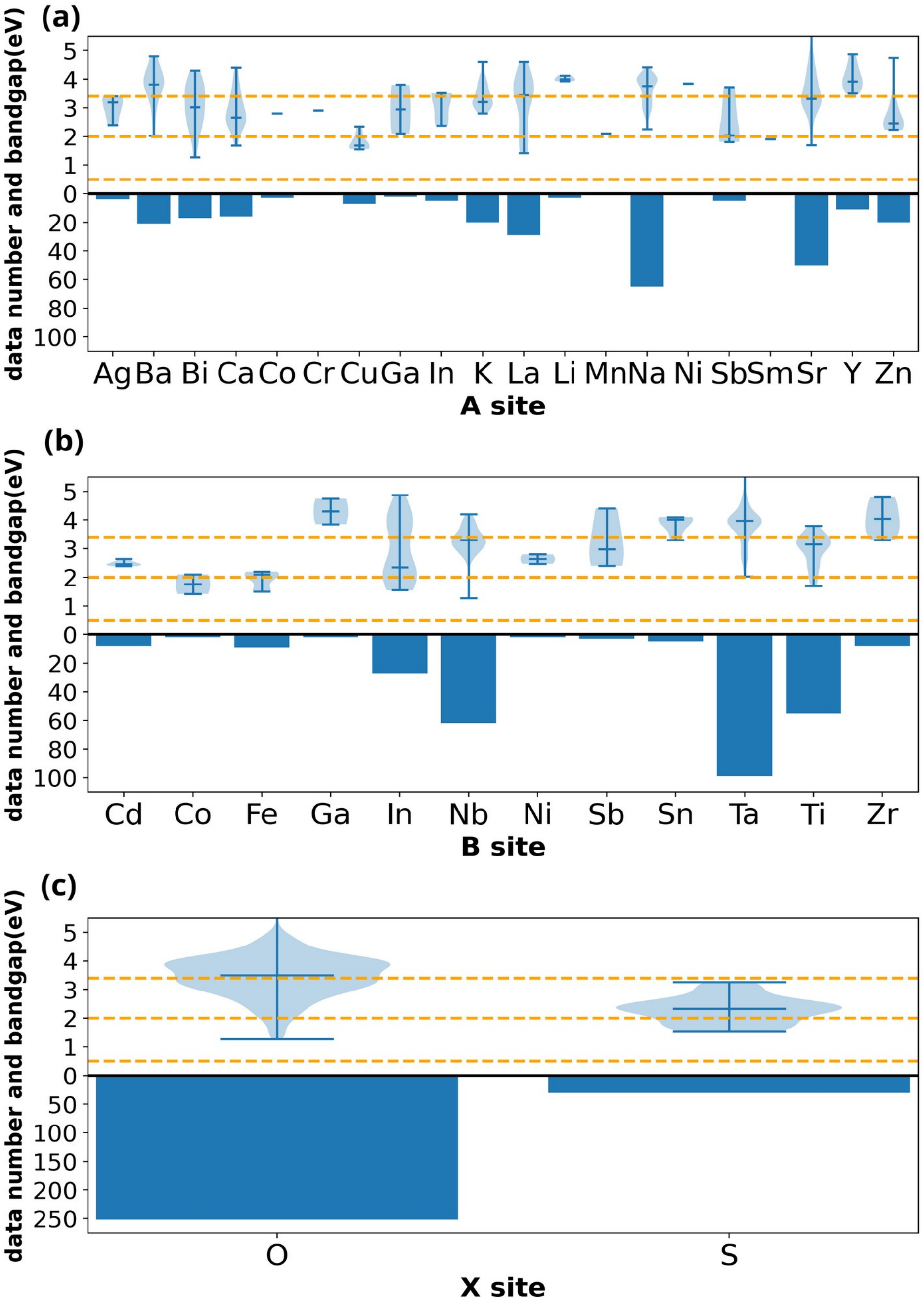

Figure 5S: Visualization of literature data. (a)A site element, (b) B site element, (c) X site element against data number and bandgap.
